# Resilience, personal recovery, and quality of life for psychiatric in-patients prior to hospital discharge: demographic and clinical determinants

**DOI:** 10.3389/fpsyt.2025.1494493

**Published:** 2025-05-23

**Authors:** Ernest Owusu, Wanying Mao, Reham Shalaby, Hossam Eldin Elgendy, Belinda Agyapong, Ejemai Eboreime, Mobolaji A. Lawal, Nnamdi Nkire, Carla T. Hilario, Yifeng Wei, Peter Silverstone, Pierre Chue, Xin-Min Li, Wesley Vuong, Arto Ohinmaa, Valerie Taylor, Andrew J. Greenshaw, Vincent I. O. Agyapong

**Affiliations:** ^1^ Department of Psychiatry, University of Alberta, Edmonton, AB, Canada; ^2^ Department of Psychiatry, Dalhousie University, Halifax, NS, Canada; ^3^ School of Nursing, University of British Columbia, Okanagan, BC, Canada; ^4^ Alberta Health Services, Addiction and Mental Health Services, Edmonton, AB, Canada; ^5^ School of Public Health, University of Alberta, Edmonton, AB, Canada; ^6^ Department of Psychiatry, Cumming School of Medicine, University of Calgary, Calgary, AB, Canada

**Keywords:** personal recovery, resilience, mood disorders, substance use disorder, schizophrenia

## Abstract

**Introduction:**

Patients with mental health challenges often see the transition from hospital to community as a test of resilience and a potential threat to recovery. Many question their ability to cope with everyday challenges. This paper examines how demographic and clinical factors predict resilience, personal recovery, and quality of life.

**Methods:**

Data were collected from psychiatric inpatients before discharge using REDCap, an online survey platform. Resilience, recovery, and quality of life were assessed with the Brief Resilience Scale (BRS), Recovery Assessment Scale (RAS), and EQ-Visual Analogue Scale (EQ-VAS). ANCOVA was used to compare group relationships. Demographic and clinical variables such as age, gender, ethnicity, and mental health diagnosis were independent variables.

**Results:**

Males had significantly higher resilience scores than females (Mdiff = 0.270, p<.001) and others (Mdiff = 0.470, p<.001). Self-identified Black individuals had higher quality of life scores than Caucasians (Mdiff = 8.79, p<.001) and Indigenous individuals (Mdiff = 14.50, p<.001). Participants with depression had significantly lower recovery scores compared to those with bipolar disorder (Mdiff = -10.25, p<.001), schizophrenia (Mdiff = -8.60, p<.001), and substance use disorder (Mdiff = -8.30, p<.005).

**Conclusion:**

Results suggest that women, younger adults, Indigenous peoples, and individuals with depression struggle more with adapting to post-discharge life. Policymakers should implement programs that focus on supporting resilience in these vulnerable groups.

## 1 Introduction

Transitions from inpatient mental healthcare to community care can be especially challenging for individuals with mental health issues, with these challenges typically categorized into personal and systemic factors (1, 2). These transitions can be perceived as tests of resilience, threatening recovery and causing anxiety about daily life challenges (3). As a result, some inpatients may relapse even before discharge (4). Addressing these concerns, such as housing, job security, and income, is essential for a successful transition (5). Systemic challenges include the risk of readmission, availability of continuity of care, suicide risks, medication management, and poor communication with community structures (6, 7). Additionally, discharge processes may be delayed due to issues such as securing community support, funding, and family factors (4). Building resilience during discharge planning can mitigate these challenges, improving post-discharge recovery outcomes (8), thereby enhancing quality of lives of patients in the community.

**Figure 1:**
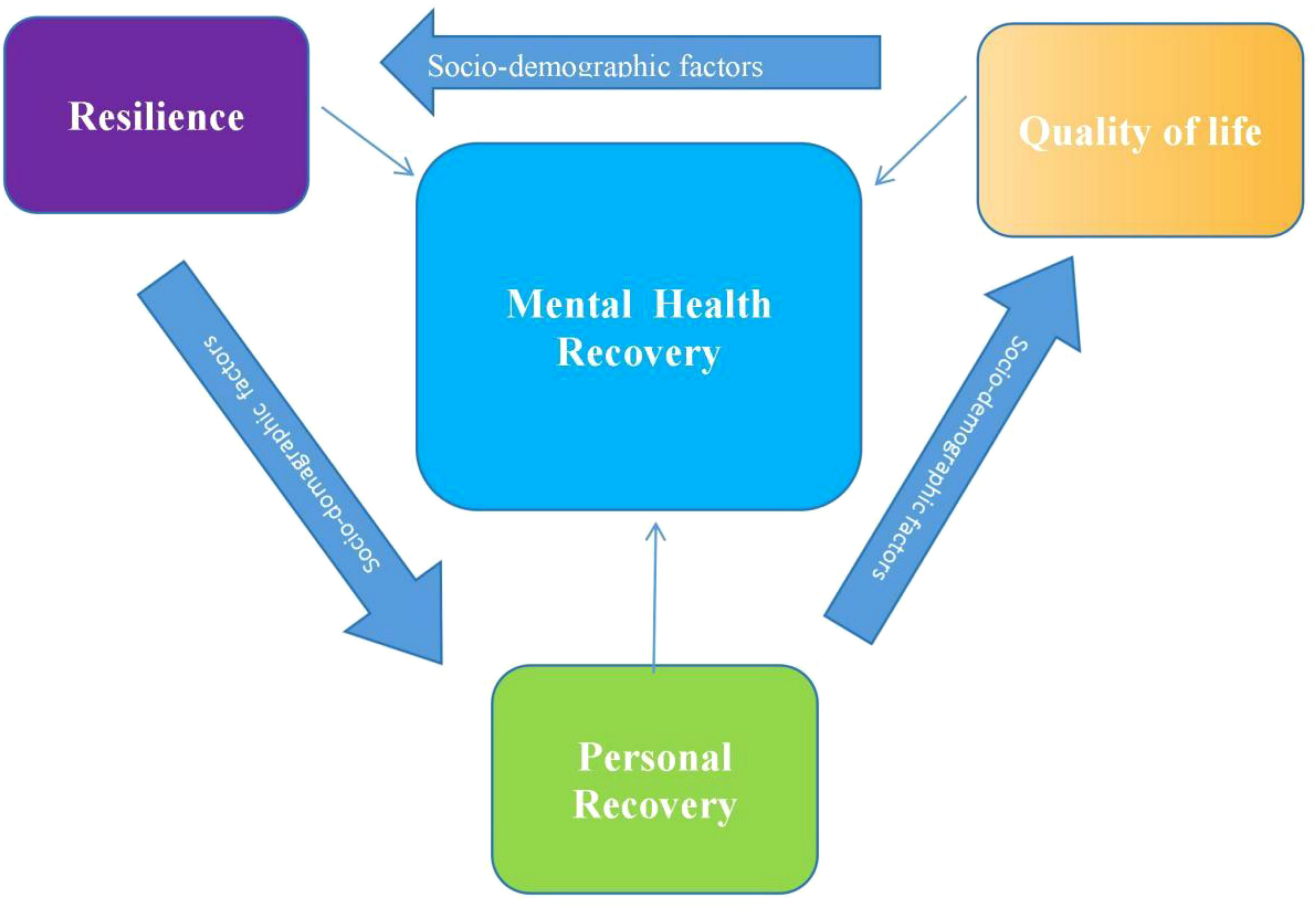
Illustration of the main conceptual framework for resilience, personal recovery, and quality of life as core contructs of mental health recovery.

The theoretical framework of this paper as illustrated in (**Figure 1**) emphasizes the connection between resilience, personal recovery, and quality of life, all influenced by demographic and clinical factors (9, 10). Understanding these factors allows healthcare professionals to tailor interventions to improve recovery outcomes. This framework acknowledges how both individual and systemic factors affect resilience and overall recovery (11, 12). Resilience, defined as the ability to adapt to adversity, is critical in mental health recovery. Research suggests resilience is a protective factor against depression and other mental disorders, fostering the ability to cope with stress (8, 13). Personal recovery, in contrast to symptom remission, emphasizes social inclusion, self-determination, and hope (14). It is a process of finding meaning in life beyond mental illness and trauma, focusing on personal goals and aspirations (14–16). Personal recovery differs from clinical recovery, as it centers on empowerment, hope, and self-directed goals (16, 17). Quality of life is perceived satisfaction in mental, physical, and social domains, which is shaped by individual goals and societal values (18).

Research has shown resilience positively impacts recovery outcomes (19, 20), with resilient individuals managing mental health challenges better, adapting to stress, and having a higher perceived quality of life (21, 22). Personal recovery, focusing on self-empowerment, also enhances resilience and quality of life (23, 24). Positive psychiatry, which integrates resilience, coping skills, and social engagement, supports the idea that resilience helps individuals find meaning and adapt after mental illness (25, 26).

In Alberta, Canada, Alberta Health Services (AHS) emphasizes the importance of stable living conditions for recovery, with housing insecurity potentially hindering recovery progress (26). Programs like “My Recovery Plan” (MRP) focus on resilience-building and meaning in life, which can improve quality of life and reduce readmissions (26, 27). Research indicates that uncoordinated discharge planning leads to higher readmission rates, as individuals may feel unsupported (28, 29). For example, Alberta’s long wait times for mental health services contribute to feelings of neglect and may lead to relapse and readmission (30).

Sociodemographic factors, such as gender, age, and ethnicity, influence resilience, recovery, and quality of life (31, 32) Gender roles impact how individuals cope with mental health challenges, with studies suggesting that women are more likely to seek social support, potentially giving them a resilience advantage (33, 34). Age also plays a role in resilience, with younger individuals facing challenges related to emerging adulthood, while older individuals may contend with physical health issues, which can affect recovery (35, 36). Mental health diagnosis significantly impacts recovery trajectories, with conditions like depression, anxiety, schizophrenia, and bipolar disorder presenting unique challenges (30, 37). Research suggests that males may have higher resilience and recovery scores than females, and that individuals over 65 tend to be more resilient than younger individuals (38, 39). Ethnic disparities also influence recovery, with Caucasians generally experiencing higher resilience and recovery compared to Black, Asian, and Indigenous individuals due to systemic barriers and discrimination (40, 41).

Research on resilience factors in mental health disorders has highlighted that conditions such as anxiety, depression, and schizophrenia are affected by individual resilience levels (42, 43). Resilience factors, like perceived ability to cope with life stressors, help individuals thrive and find meaning in their experiences (44, 45). Healthcare workers’ understanding of vulnerability and resilience factors can improve interventions and support for individuals transitioning to community care (8, 29). Ongoing research into these factors is vital for developing evidence-based practices that foster recovery.

In mental health diagnoses, depression often correlates with lower resilience and poorer recovery outcomes compared to conditions like schizophrenia or bipolar disorder (46, 47). Patients with depression may face more challenges in recovery due to the nature of their condition, which affects their ability to adapt and recover post-discharge (48, 49).

There remain gaps in the literature about how gender, age, ethnicity, and clinical diagnosis impact resilience, recovery, and quality of life after discharge. Understanding the complex factors that influence resilience, personal recovery, and quality of life is crucial for improving outcomes for individuals transitioning from inpatient psychiatric care to community care.

To address gaps in the literature, this study seeks to assess resilience, personal recovery, and quality of life levels using the Brief Resilience Scale (BRS), Recovery Assessment Scale (RAS), and EQ-VAS respectively (13, 50, 51). The study seeks to explore the relationship between sociodemographic and clinical characteristics and how they impact resilience, personal recovery, and quality of life of patients who are ready to be discharged from inpatient psychiatric units in Alberta.

## 2 Methodology

### 2.1 Study setting and design

This study was conducted in Alberta, Canada, with a population of 4.7 million (52). In 2018, there were 28,571 adult discharges from psychiatric inpatient units across Alberta (4, 28). Participants were recruited from ten acute mental health units across Edmonton, Calgary, and Grand Prairie using a pragmatic stepped-wedge cluster-randomized design. The study aimed to evaluate the impact of supportive text messages (Text4Support) and peer support services (PSS) on readmission rates for patients with mental illness discharged from acute psychiatric hospitals. The main study protocol is available online (4, 28).

### 2.2 Sample size calculation

Using a projected margin of error of 3 for the mean EQ-VAS, a 95% confidence level, a population of 28,571, and a population variance of 2000, an online script (53) estimated a required sample size of 829. This estimation ensures an adequate number of participants to minimize errors while avoiding an excessively large sample size (54). For the ANCOVA, the observed power for the BRS, RAS and EQ-VAS were 0.99 (99%), 1.00 (100%), 1.00 (100%) respectively at alpha 0.05.

### 2.3 Ethics statement

The study was approved by the University of Alberta Health Ethics Research Board (Ref Pro00111459) and received additional approval from the regional health authority. Ethical approval was obtained for verbal consent to interviews and implied consent for electronic surveys. Written informed consent was obtained from all participants to access their health records.

### 2.4 Data collection

Data were collected through REDCap (55), an online platform, as part of an ongoing clinical trial examining the utility of Text4Support and Peer Support in reducing inpatient readmission rates in Alberta (28). Eligible participants were adults (18+), diagnosed with any mental health condition, ready for discharge, had a mobile device, could read English, and could provide informed consent. Sociodemographic data (age, gender, ethnicity, education, relationship status, employment) and clinical data (diagnosis, admission duration) were collected. The study data were gathered from March 8, 2022, to November 5, 2023. Baseline surveys were administered with the assistance of research team members after written consent. Phone and healthcare numbers served as primary identifiers.

### 2.5 Outcome measures

Primary outcomes included scores on the BRS, RAS, and EQ-VAS, and the association between these scores and demographic/clinical factors like gender, age, ethnicity, and mental health diagnosis.

The BRS evaluates resilience, defined as the ability to recover from stress (13). It comprises six statements, with responses ranging from 1–5 on a Likert scale, yielding a score between 6–30. Resilience levels are categorized as low (1.00–2.99), normal (3.00–4.30), and high (4.31–5.00) (4). For this analysis, normal and high resilience were grouped into one category (3.00–5.0). The BRS demonstrated good internal consistency (Cronbach α = 0.84) and reliability (r = 0.67) (56, 57). In a related study, the BRS showed good reliability (α = 0.84) and validity (r = 0.80, p <.001) (58).

The RAS is a 24-item scale that assesses recovery perceptions on a 5-point Likert scale (42). It includes five factors: (1) personal confidence and hope, (2) willingness to ask for help, (3) goal orientation, (4) reliance on others, and (5) no domination by symptoms (59). The RAS shows excellent psychometric properties (α = 0.94) and test-retest reliability (r = 0.88) (50, 60). The total score ranges from 24–120, with higher scores correlating with better quality of life and empowerment, while lower scores reflect more severe symptoms.

The EQ-VAS measures quality of life by assessing perceived health status on a scale from 0 (worst) to 100 (best) (51). It has proven to be a reliable and valid tool with test-retest reliability ranging from 0.65 to 0.91 (61–63).

### 2.6 Statistical analysis

Statistical analysis was conducted using SPSS for Mac, version 25 (IBM) (64). Baseline characteristics (sociodemographic, clinical) were summarized by age groups (<=25, 26–40, >40 years) as numbers, percentages, or means with standard deviations. Multiple ANCOVAs compared the relationship between groups, with self-rated BRS, RAS, and EQ-VAS scores as dependent variables investigated separately. The independent variables included demographic/clinical factors like age, gender, ethnicity, and diagnosis. Covariates included other demographic or clinical factors not designated as independent variables. Assumptions of ANCOVA were checked (linearity, normality, homogeneity). A *post-hoc* Tukey’s HSD test assessed group differences. Missing data were not imputed, and significance was set at p < 0.05.

Skewness and kurtosis were calculated to assess the normality of the data distribution. The results were as follows

- BRS: skewness = -0.33, kurtosis = 0.00 (standard error of skewness = 0.08, standard error of kurtosis = 0.15),
- RAS: skewness = -0.33, kurtosis = 0.74 (standard error of skewness = 0.08, standard error of kurtosis = 0.15),
- EQ-VAS: skewness = 0.48, kurtosis = 0.31 (standard error of skewness = 0.08, standard error of kurtosis = 0.16).

Based on the skewness values falling between -2 and 2 and kurtosis values falling between -7 and 7, the data were deemed to follow a normal distribution.

## 3 Results

**Table 1** outlines the sociodemographic distribution of participants. Among the 1,004 participants, 35.8% were 25 years or younger, 34.7% were between 26 and 40 years, and 29.5% were over 40 years. Most participants identified as female (54.8%), with 42.4% identifying as male. Ethnically, 62.4% were Caucasian, and 51.4% had completed high school. The majority were single (58.9%), unemployed (53.4%), and rented their homes (32.3%). Over 26% had a clinical diagnosis of depression. The mean scores were: BRS 2.81 (SD=0.83), RAS 90.21 (SD=15.42), and EQ-VAS 67.79 (SD=20.12). For the ANCOVA, the observed power for the BRS, RAS and EQ-VAS were 0.99 (99%), 1.00 (100%), 1.00 (100%) respectively at alpha 0.05.

**Table 1:** Distribution of participants’ sociodemographic and clinical characteristics across age categories, entries are means ± (SD).

Variables, N (%)	<= 25 years N =360	26–40 years N =348	>40 years N =296	Total N =1004
Gender				
Male Female Other Gender	141 (39.2%) 202 (56.1%) 17 (4.7%)	156 (44.8%) 181 (52.0%) 11 (3.2%)	129 (43.6 167 (56.4%) 0 (0.0%)	426 (42.4%) 550 (54.8%) 28 (2.8%)
Ethnicity Categories				
Caucasians Indigenous People Black People Asians Other	191 (53.1%) 39 (10.8%) 52 (14.4%) 55 (15.3%) 23 (6.4%)	221 (63.5%) 28 (8.0%) 37 (10.6%) 34 (9.8%) 28 (8.0%)	213 (72.0%) 28 (9.5%) 14 (4.7%) 22 (7.4%) 19 (6.4%)	625 (62.3%) 95 (9.5%) 103 (10.3%) 111 (11.1%) 70 (7.0%)
Education Categories				
Less than High School High School Diploma Post-Secondary Education Other	13 (3.6%) 262 (72.8%) 72 (20.0%) 13 (3.6%)	17 (4.9%) 151 (43.4%) 175 (50.3%) 5 (1.4%)	9 (3.0%) 103 (34.6%) 170 (57.4%) 14 (4.7%)	39 (3.9%) 516 (51.4%) 417(41.7%) 32 (3.2%)
Current Relationship Status				
Single Separated/Divorced Partnered/Married/Common law. Widowed Other	273 (75.8%) 0 (0.0%) 65 (18.1%) 0 (0.0%) 22 (6.1%)	216 (62.1%) 23 (6.6%) 103 (29.6%) 3 (0.9%) 3 (0.9%)	102 (34.5%) 57 (19.3%) 124 (41.9%) 7 (2.4%) 6 (2.0%)	591(58.9%) 80 (8.0%) 292 (29.1%) 10 (1.0%) 31 (3.1%)
Current Employment Status				
Student Employed Unemployed Retired Other	68 (18.9%) 80 (22.2%) 194 (53.9%) 0 (0.0%) 16 (5.0%)	8 (2.3%) 122 (35.1%) 207 (59.5%) 3 (0.9%) 8 (2.3%)	1 (0.3%) 96 (32.4%) 135 (45.6%) 55 (18.6%) 9 (3.0%)	77 (7.7%) 298 (29.7%) 536 (53.4%) 58 (5.8%) 35 (3.5%)
Current Housing Status				
Own Home Rented Accommodation Live with Family/Friends Couch/Shelter/Street/Other	16 (4.4%) 72 (20.0%) 252 (70.0%) 20 (5.6%)	62 (17.8%) 134 (38.5%) 128 (36.8%) 24 (6.9%)	12 7(42.9%) 118 (39.9%) 32 (10.8%) 19 (6.4%)	205 (20.4%) 324 (32.3%) 412 (41.0%) 63 (6.3%)
Primary MH Dx				
Depression Bipolar Disorder Anxiety Disorder Schizophrenia Personality Disorder Substance Use Disorder Other	90 (25.0%) 62 (17.2%) 52 (14.4%) 57 (15.8%) 58 (16.1%) 12 (3.3%) 29 (8.1%)	77 (22.1%) 84 (24.1%) 38 (10.9%) 70 (20.1%) 30 (8.6%) 18 (5.2%) 31 (8.9%)	95 (32.1%) 60 (20.3%) 45 (15.2%) 34 (11.5%) 3 (1.0%) 21 (7.1%) 38 (12.8%)	262 (26.1%) 206 (20.5%) 135 (13.4%) 161 (16.0%) 91 (9.1%) 51 (5.1%) 98 (9.8%)
Variables	< =25 years	26–40 years	>40 years	Total
	Mean scores (SD)			
BRS	2.67 (0.79)	2.90 (0.82)	2.90 (0.82)	2.81 (0.83)
RAS	87.60 (14.98	92.43 (15.20)	90.80 (15.83)	90.21 (15.42)
EQ-VAS	65.93 (20.32)	69.43 (19.70)	68.11 (20.31)	67.79 (20.12)

**Table 2** presents the ANCOVA results for four covariates (gender, age, ethnicity, and mental health diagnosis). Statistically significant differences in BRS, RAS, and EQ-VAS scores were observed across different age, gender, ethnicity, and mental health diagnosis groups, after adjusting for other demographic and clinical factors. Gender was significantly associated with all three scores: RAS (F(2,992)=5.85, p=0.003), BRS (F(2,994)=15.30, p<0.001), and EQ-VAS (F(2,981)=6.89, p=0.001). Age was linked to both RAS (F(2,992)=6.45, p=0.002) and BRS (F(2,994)=8.83, p<0.001), but not EQ-VAS (F(2,981)=0.682, p=0.177). Ethnicity significantly influenced all three scores: RAS (F(4,990)=8.13, p<0.001), BRS (F(4,992)=9.30, p<0.001), and EQ-VAS (F(4,979)=8.10, p<0.001). Mental health diagnosis also significantly impacted all scores: RAS (F(6,998)=13.14, p<0.001), BRS (F(6,990)=18.23, p<0.001), and EQ-VAS (F(6,977)=4.27, p<0.001).

**Table 2:** Results of four ancova analysis.

Variable	df	Brief Resilience Scale (BRS)				Recovery Assessment Scale (RAS)				EQ-VAS			
		Adjusted Mean (CI)	F	P	Partial Eta Sq	Adjusted Mean (CI)	F	P	Partial Eta Sq	Adjusted Mean (CI)	F	P	Partial Eta Sq
Gender													
Male Female Other	2	2.97 (2.89-3.04) 2.70 (2.63-2.76) 2.50 (2.20-2.80)	15.30	0.00	0.03	92.10 (90.64-93.50) 88.92 (87.64-90.21) 86.60 (80.89-92.23)	5.85	.003	.012	70.30(68.40-72.22) 66.22(64.53-67.92) 60.10(52.64-67.50)	6.89	.001	.014
Age Cat.													
< 25 years 26–40 years **>**40 years	2	2.65 (2.56-2.74) 2.88 (2.80-2.97) 2.90 (2.80-3.00)	8.83	0.00	0.02	88.13 (86.41-89.80) 92.35 (90.76-93.94) 90.24(88.34-92.14)	6.45	.002	.013	66.55(64.31-68.80) 69.34(67.25-71.44) 67.45(64.92-69.98)	682.01	.177	.004
Ethnicity.													
Caucasians Indigenous People Black People Asians Other	4	2.30(2.63-2.80) 2.93(2.77-3.10) 3.11(2.95-3.30) 3.90(2.75-3.04) 3.10(2.88-3.30).	9.30	0.00	.036	88.44(87.25-89.63) 90.80 87.75-93.86) 96.49 (93.53-99.44) 90.81(87.97-93.64) 95.10 (91.52-98.63)	8.13	<.001	.032	66.65(65.07-68.22) 60.95(56.92-64.97) 75.44(71.55-79.33) 71.69(67.97-75.40) 69.78(65.10-74.52)	8.10	<.001	.032
Primary MH Dx													
Depression Bipolar Disorder Anxiety Dx Schizophrenia Personality Dx S U Disorder Other	6	2.50 (2.40-2.40) 2.99 (2.89-3.10) 2.69 (2.60-2.83) 3.17 (3.05-3.30) 2.65 (2.49-2.82) 3.20 (2.99-3.41) 2.74 (2.60-2.89)	18.32	0.00	0.10	85.30 (83.50-87.10) 95.52 (93.51-97.53) 90.40 (8792-92.90) 93.87(91.54-96.19) 85.10 (81.95-88.26) 93.56 (89.51-97.60) 88.90 (85.97-91.83)	13.14	<.001	.074	63.02(60.60-65.44) 71.80(69.06-74.54) 68.23(64.86-71.60) 68.70(65.60-71.84) 67.41(63.11-71.72) 71.45(66.00-76.90) 68.46(64.52-72.41)	4.27	<.001	.026

Given these significant differences, pairwise comparisons of BRS, RAS, and EQ-VAS scores were performed using Tukey’s HSD. **Table 3** summarizes these findings. Regarding gender, males scored higher in BRS, EQ-VAS, and RAS than females. In terms of age, participants under 26 had lower resilience scores compared to those between 26-40 (Mdiff = -0.241) and over 40 years (Mdiff = -0.257). Participants aged 26–40 also had significantly higher recovery scores than those under 26 (Mdiff = 4.25).

**Table 3:** Results of *Post Hoc* analysis for gender, age, and ethnicity.

Variable		Brief Resilience Scale (BRS)			Recovery Assessment Scale (RAS).			EQ VAS		
		Mean difference	CI (95%)	P	Mean difference	CI (95%)	P	Mean difference	CI (95%)	P
Gender										
Male	Female	0.270	(.144) - (. 397)	.000	3.18	(.775) – (5.58)	.005	4.10	(.909) – (7.24)	.006
	Other	0.470	(.093) – (.846)	.000	5.54	(-1.61) – (12.70)	.191	10.23	(.848) – (19.61)	.027
Female	Male	-0.270	(-.397)- (-.144)	.000	-3.18	(-5.58) – (-.775)	.005	-4.10	(-7.24) – (-.909)	.006
	Other	0.199	(-.175) -(.573)	.605	2.36	(-4.74) – (9.47)	1.00	6.15	(-3.16) – (15.46)	.340
Other	Male	-0.470	(-.846)- (-.093)	.009	-5.54	(-12.70) – (1.61)	.191	-10.23	(-19.61) – (-.648)	.027
	Female	-0.199	(-.573) -(.175)	.605	-2.36	(-9.47) – (4.74)	1.00	-6.15	(-15.46) – (3.16)	.340
Age Cat.										
	26–40 years	-0.241	(-.392)- (-.091)	.000	-4.25	(-7.10) – (-1.39)	.001			
	**>**40 years	-0.257	(-.434) -(-.081)	.001	-2.14	(-5.48) – (1.21)	.381			
26–40 years	< 25 years	-0.241	(.091) -(.392)	.000	4.25	(1.39) – (7.10)	.001			
	**>**40 years	-0.016	(-.176) -(.143)	1.00	2.11	(-.908) – (5.13)	.281			
	< 25 years	0.257	(.081) -(.434)	.001	2.14	(-1.21) – (5.48)	.381			
	26–40 years	0.016	(-.143) -(.176)	1.00	-2.11	(-5.13) – (.908)	.281			
Ethnicity.										
Caucasians	Indigenous people	-0.237	(-.485) – (.011)	.072	-2.37	(-7.07) – (2.34)	1.00	5.70	(-.502) – (11.89)	.099
	Black people	-0.415	(-.658) – (-.172)	.000	-8.05	(-12.64) – (-3.45)	**<**.001	-8.79	(-14.85) – (-2.72)	**<**.001
	Asians	-0.203	(-.437) – (.031)	.002	-2.40	(-6.80) – (2.05)	1.00	-5.04	(-10.85) – (.767)	.148
	Other	-0.203	(-.659) -(-.093)	.002	-6.64	(-12.03) – (-1.25)	.005	-3.14	(-10.29) – (4.02)	1.00
Indigenous People	Caucasians	0.237	(-.011)- (.485)	.072	2.37	(-2.34) – (7.07)	1.00	-5.60	(-11.89) – (.502)	.099
	Black people	-0.178	(-.499) – (.143)	1.00	-5.68	(-11.77) – (.408)	.088	-14.48	(-22.51) – (-6.45)	<.001
	Asians	0.034	(-282) – (.350)	1.00	-.009	(-5.99) – (5.98)	1.00	-10.74	(-18.61) – (-2.86)	.001
	Other	-0.139	(-.493) – (.215)	1.00	-4.28	(-11.01) – (2.46)	.745	-8.83	(-17.76) – (.094)	.055
Black People	Caucasians	-415	(.172) – (.658)	.000	8.05	(3.45) – (12.65)	**<**.001	8.79	(2.72) – (14.85)	**<.**001
	Indigenous	0.178	(-.143) – (.499)	1.00	5.68	(-.408) – (11.77)	.088	14.50	(6.45) – (22.51)	**<**.001
	Asians	0.212	(-.095) – (.520)	.526	5.68	(-.138) – (11.49)	.061	3.74	(-3.89) – (11.38)	1.00
	Other	0.390	(-.309) – (.387)	1.00	1.41	(-5.20) – (8.02)	1.00	5.65	(-3.11) – (14.41)	.700
Asians	Caucasians	0.203	(-.031) – (.437)	.149	2.40	(-2.05) – (6.80)	1.00	5.04	(-.767) – (10.85)	.148
	Indigenous people	-0.034	(-.350) – (.282)	1.00	.099	(-5.98) – (5.99)	1.00	10.74	(2.86) – (18.61)	.001
	Black people	-0.212	(-.520) – (.095)	.526	-5.67	(-11.49) – (.138)	.061	-3.75	(-11.38) – (3.89)	1.00
	Other	-0.173	(-.516) – (.169)	1.00	-4.27	(-10.76) – (2.23)	.652	1.91	(-6.69) – (10.51)	1.00
Other	Caucasian	-0.376	(.093) – (.659)	.002	6.64	(1.25) – (12.02)	.005	3.14	(-4.02) – (10.29)	1.00
	Indigenous people	0.139	(-.215) – (.493)	1.00	4.30	(-2.46) – (11.01)	.745	8.83	(-.094) – (17.76)	.055
	Black people	-0.039	(-.387) – (.309)	1.00	-1.41	(-8.02) – (5.20)	1.00	-5.65	(-14.41) – (3.11)	.700
	Asian	0.173	(-.169) – (.516)	1.00	4.30	(-2.23) – (10.76)	.652	-1.91	(-10.51) – (6.69)	1.00

Ethnicity comparisons revealed that Caucasians had lower resilience than Black participants (Mdiff = -0.415), Asians (Mdiff = -0.203), and others (Mdiff = -0.203). Black participants had higher quality-of-life scores compared to Caucasians (Mdiff = 8.79), and Asians scored better than Indigenous participants (Mdiff = 10.74). In terms of recovery, Black participants scored higher than Caucasians (Mdiff = 8.05).

**Table 4** shows the pairwise comparison for mental health diagnoses. Participants with depression had significantly lower resilience compared to those with bipolar disorder (Mdiff = -0.505), schizophrenia (Mdiff = -0.680), and substance use disorder (Mdiff = -0.713). Depression also correlated with lower quality of life and recovery compared to bipolar disorder, schizophrenia, and substance use disorder. Participants with bipolar disorder had better resilience and recovery than those with anxiety, personality disorder, and others. Additionally, individuals with schizophrenia had higher resilience and recovery compared to those with personality disorders. Participants with substance use disorder had lower resilience than those with schizophrenia (Mdiff = 0.463).

**Table 4:** Results of *post hoc* analysis for mental health diagnosis.

Variable		BRS scores			RAS scores			EQ VAS scores		
		Mean difference	CI (95%)	P	Mean difference	CI (95%)	P	Mean difference	CI (95%)	P
Primary MH Dx										
Depression	Bipolar Dis.	-0.505	(-.723) – (-.290)	.000	-10.25	(-14.40) – (-6.09)	<.001	-8.78	(-14.42) – (-3.14)	<.001
	Anxiety Dis.	-0.207	(-.454) – (.040)	.226	-5.13	(-9.88) – (-.381)	.022	-5.21	(-11.63) – (1.21)	.286
	Schizophrenia	-0.680	(-.920) – (-.440)	.000	-8.60	(-13.19) – (-3.99)	<.001	-5.68	(-11.90) – (.533)	.115
	Personality Dis	-0.164	(-.458) – (.129)	1.00	.170	(-5.48) – (5.82)	1.00	-4.40	(-12.10) – (3.30)	1.00
	S U Disorder	-0.713	(-1.070) – (-.355)	.000	-8.30	(-15.14) – (-1.42)	.005	-8.44	(-17.69) – (.815)	.117
	Other	-0.250	(-.529) – (.029)	.135	-3.63	(-8.98) – (1.72)	.820	-5.45	(-12.66) – (1.76)	.454
Bipolar Disorder	Depression	0.506	(.290) – (.723)	.000	10.25	(6.09) – (14.40)	<.001	8.78	(3.14) – (14.42)	<.001
	Anxiety Dis.	0.299	(.041) – (.557)	.009	5.12	(.153) – (10.07)	.037	3.57	(-3.15) – (10.29)	1.00
	Schizophrenia	-0.174	(-.424) – (.076)	.724	1.66	(-3.14) – (6.45)	1.00	3.10	(-3.41) – (9.61)	1,00
	Personality Dis	0.342	(.038) – (.646)	.013	10.42	(4.57) – (16.25)	<.001	4.40	(-3.57) – (12.35)	1.00
	S U Disorder	-0.206	(-.572) – (.159)	1.00	1.96	(-5.04) – (8.96)	1.00	.348	(-9.11) – (9.80)	1.00
	Other	0.256	(-.032) – (.545)	.144	6.62	(1.08) – (12.14)	.006	3.34	(-4.13) – (10.80)	1.00
Anxiety Disorder	Depression	0.207	(-.404) – (.454)	.226	5.13	(.381) – (9.88)	.022	5.21	(-1.21) – (11.63)	.286
	Bipolar Dis.	-0.299	(-.557) – (-.041)	.009	-5.12	(-10.07) – (-.153)	.037	-3.57	(-10.29) – (3.15)	1.00
	Schizophrenia	-0.473	(-.750) – (-.196)	.000	-3.46	(-8.78) – (1.86)	1.00	-.470	(-7.67) – (6.72)	1.00
	Personality Dis	0.043	(-.279) – (.364)	1.00	5.30	(-.892) – (11.49)	.194	.817	(-7.61) – (9.24)	1.00
	S U Disorder	-0.505	(-.891) – (-.120)	.001	-3.15	(-10.55) – (4.24)	1.00	-3.22	(-13.19) – (6.75)	1.00
	Other	-0.043	(-.354) – (.268)	1.00	1.50	(-4.47) – (7.47)	1.00	-.234	(-8.29) – (7.82)	1.00
Schizophrenia	Depression	0.680	(.440) – (.920)	.000	8.60	(3.99) – (13.19)	<.001	5.50	(-.533) – (11.90)	.115
	Bipolar Dis.	0.174	(-.076) – (.424)	.724	-1.66	(-6.45) – (3.14)	1.00	-3.10	(-9.61) – (3.41)	1.00
	Anxiety Dis.	0.473	(.196) – (.750)	.000	3.46	(-1.86) – (8.78)	1.00	.470	(-6.72) – (7.67)	1.00
	Personality Dis	0.516	(.197) – (.835)	.000	8.76	(2.62) – (14.90)	**<**.001	1.30	(-7.06) – (9.64)	1.00
	S U Disorder	-0.033	(-.408) – (.342)	1.00	.307	(-6.88) – (7.50)	1.00	-3.22	(-12.45) – (6.94)	1.00
	Other	0.430	(.130) – (.731)	.000	4.96	(-.800) – (10.72)	.186	-.234	(-7.53) – (8.00)	1.00
Personality Dis	Depression	0.164	(-.129) – (.458)	1.00	-.170	(-5.82) – (5.48)	1.00	4.40	(-3.30) – (12.10)	1.00
	Bipolar Dis.	-0.342	(-.464) – (-.038)	.013	-10.42	(-16.25) – (-4.57)	<.001	-4.40	(-12.35) – (3.57)	1.00
	Anxiety Dis.	-0.043	(-.364) – (.279)	1.00	-5.30	(-11.49) – (.892)	.194	-.817	(-9.24) – (7.61)	1.00
	Schizophrenia	-0.516	(-.835) – (-.197)	.000	-8.76	(-14.90) – (-2.62)	**<**.001	-1.30	(-9.64) – (7.06)	1.00
	S U Disorder	-0.548	(-.969) – (-.127)	.002	-8.46	(-16.54) – (-.369)	.031	-4.04	(-14.99) – (6.91)	1.00
	Other	-0.086	(-.435) – (.264)	1.00	-3.80	(-10.52) – (2.92)	1.00	-1.05	(-10.18) – (8.07)	1.00
S U Disorder	Depression	0.713	(.355) – (1.070)	.000	8.30	(1.42) – (15.14)	.005	8.44	(-.815) – (17.68)	.117
	Bipolar Dis.	0.206	(-.159) – (.572)	1.00	-1.96	(-8.96) - (5.04)	1.00	-.348	(-9.80) – (9.11)	1.00
	Anxiety Dis.	0.505	(.120) – (.891)	.001	3.15	(-4.24) – (10.55)	1.00	3.22	(-6.75) – (13.19)	1.00
	Schizophrenia	0.033	(-.342) – (.408)	1.00	-.307	(-7.50) – (6.88)	1.00	2.80	(-6.94) – (12.45)	1.00
	Personality Dis	0.548	(.127) – (.969)	.002	8.45	(.369) – (16.54)	.031	4.04	(-6.91) – (14.99)	1.00
	Other	.463	(.060) – (.866)	.010	4.66	(-3.07) – (12.38)	1.00	2.98	(-7.42) – (13.39)	1.00
Other	Depression	0.250	(-.029) – (.529)	.135	3.63	(-1.72) – (8.98)	.820	5.45	(-1.76) – (12.66)	.454
	Bipolar Dis.	-0.256	(-.545) – (.032)	.144	-6.62	(-12.14) – (-1.08)	.006	-3.34	(-10.80) – (4.13)	1.00
	Anxiety Dis.	0.043	(-.268) – (.354)	1.00	-1.50	(-7.47) – (4.47)	1.00	-.236	(-7.82) – (8.29)	1.00
	Schizophrenia	-0.430	(-.731) – (-.130)	.000	-4.96	(-10.72) – (.800)	.186	-.236	(-8.00) – (7.53)	1.00
	Personality Dis	0.086	(-.264) – (.435)	1.00	3.80	(-2.92) – (10.52)	1.00	1.10	(-8.07) – (10.18)	1.00
	S. U. Dx.	-0.463	(-.866) – (-.060)	.010	-4.66	(-12.38) – (3.07)	1.00	-2.98	(-13.39) – (7.42)	1.00

## 4 Discussion

The primary objective of this paper was to explore the relationship between sociodemographic and clinical characteristics and how they impact resilience, personal recovery, and quality of life of patients who are ready to be discharged from inpatient psychiatric units in Alberta. The study’s results reveal notable variations in resilience, personal recovery, and quality of life across different sociodemographic groups.

First, males scored significantly higher than females on resilience, recovery, and quality of life. Previous studies, including those by Bahadır (2015) (65) and Sürücü & Bacanlı (2010) (66), also report higher resilience in males, especially in personal power, initiative, and leadership. Research suggests that women may be more susceptible to the effects of childhood trauma than men (67), with resilience serving as a protective factor. Gender-sensitive approaches to resilience must consider the biological and social factors that influence men’s and women’s vulnerability to trauma and their mental health responses (68, 69). This study aligns with a study on university students that found higher resilience in male students (70). Societal gender expectations such as competitiveness for men and nurturing roles for women (71, 72) may shape resilience levels (45). Gender influences mental health responses, with men less likely to seek help due to social stigma, which can affect their recovery (73, 74).

Second, younger participants, particularly those under 26, showed lower resilience levels than older groups (26–40 and 40+), suggesting that age influences resilience. Older individuals often demonstrate greater adaptability and recovery, though resilience in younger people may be linked to emerging adulthood traits such as individualism and financial independence (75). Studies comparing resilience in young and older adults consistently find greater resilience in those over 65 (38, 75). Similarly, a UK study found that older adults were more resilient than younger adults under 26 (75).

Third, racial and ethnic differences were also evident in this study, with Black participants displaying higher resilience and recovery than their Caucasian counterparts. This suggests cultural, social, and community factors shape resilience. Research indicates that Black Canadians experience higher rates of depression, often linked to experiences of racism and discrimination (76, 77). Despite this, resilience is a key factor in improving treatment outcomes for depression (13), and Black individuals may have stronger resilience due to cultural identity and community support (78). A study on African American and Caucasian breast cancer patients found that perceived discrimination affected recovery and quality of life, with African Americans reporting lower quality of life (79). Similarly, Black participants with schizophrenia reported higher life quality than their White counterparts (80). This highlights the role of cultural identity and community engagement in resilience and recovery.

Fourth, individuals with depression had significantly lower resilience and personal recovery than those with other diagnoses, indicating the influence of mental health on these factors. Depressive disorder was associated with lower resilience and recovery compared to other mental health diagnoses, reinforcing the idea that some conditions may impede recovery and adaptation. This aligns with studies indicating lower resilience in those with depression (81, 82). Depression is often marked by hopelessness and low self-esteem, factors that contribute to lower resilience (81). Depression also correlates with higher relapse rates and poorer quality of life (48, 83, 84).

These findings underscore the complex relationships between gender, age, race, and mental health in determining resilience and recovery outcomes. Interventions may need to be designed and tailored to address the unique challenges faced by different sociodemographic groups, particularly younger individuals and those with mental health diagnoses such as depression.

## 5 Limitations

The study has several limitations. First, due to the self-report nature of the scales, patients’ responses could not be clinically validated. However, the scales used were validated, and self-reporting is practical for this type of study. Second, the study sample was drawn from a subset of a larger stepped-wedge cluster-randomized study, with an inclusion criterion of owning a mobile phone (28). This selection bias may have skewed the sample, as those without cell phones were excluded. Future studies could reduce this bias by offering alternative communication methods, such as email. Third, the lack of a control group limits the ability to draw conclusions about how the scores compare to the general population. Fourth, the study did not gather data on participants’ biological sex, preventing an analysis of its impact on resilience, recovery, and quality of life. Again, this paper did not include interaction analysis to provide the joint interaction effects for the sociodemographic and clinical characteristics to identify individuals at greatest risk of poorer outcome of resilience, personal recovery and quality of life. Future studies may examine these interaction effects. Also, while the study achieved a statistical power of 100%, which indicates a very low risk of Type II errors, this can also be considered a limitation. A power of 100% is often regarded as unrealistic or unnecessary in real-world settings, as it might suggest that the sample size was too large. Additionally, measurement invariance of the outcome measures used in this study is not well-known (56, 85, 86). Therefore, any comparisons of indicator means and covariances across demographics should be interpreted cautiously, which we acknowledge as a limitation of our study. Despite these limitations, the study provides valuable insights into how demographic and clinical factors influence resilience, recovery, and quality of life in patients discharged from inpatient psychiatric units.

## 6 Conclusions

In summary, the study outcome suggests that while there are some strengths, such as perceived recovery, there may also be areas for improvement, such as enhancing resilience and addressing the variability in health perceptions. The findings indicate that males tend to report higher resilience and quality of life compared to females, younger participants (under 26) appear to have lower resilience than older individuals, and Black participants seem to experience better resilience and recovery outcomes than Caucasians. These results may inform the development of policies and interventions aimed at improving mental health and well-being for individuals discharged from psychiatric hospitals. Programs designed to build resilience and improve recovery, such as daily supportive text messaging (87–89) and peer support services (90–92), might be helpful. The ongoing randomized trial related to this research seeks to explore the impact of these interventions on health outcomes for patients discharged from acute psychiatric hospitals. Tailoring interventions that consider sociodemographic characteristics and mental health diagnoses might enhance recovery and reduce readmission risk. Further research clarifies the long-term trajectories and factors influencing resilience, recovery, and quality of life for patients post-discharge and supports the refinement of practices that aid individuals with mental health challenges throughout their recovery. Also, future research will examine the measurement properties and invariance of these outcome measures most especially the BRS to ensure more robust and valid comparisons across diverse populations.

## Data Availability

The raw data supporting the conclusions of this article will be made available by the authors, without undue reservation.
